# 

**Published:** 1870-12

**Authors:** 


### INDEX TO VOLUME XXVI.

Argentine Albuminuria, 33.
Aconite Poisoning, 45, 384.
Arkansas Hot Springs, 161.
Aconitine Toxic Prop., 219.
Atropia Poisoning, 298, 485.
Anatomy in Maine, 299.
Anuria Prolonged, 395.
Atropia Subcutan. Inject., 550.
Asthma, Infantile, 603.
Ammonia Muriate, 603,
Amblyopia, 605.
Alcoholism, 621, 685.
Absinthic Epilepsy, 621, 685.
Arsenic in Phthisis, 681.
After-pains, Management, 684.

Bromides. Elimination of, 1.
Biatomic Metalloids, 89.
Blood Corpuscles, Prof. Freer's Discovery, 225.
Bones, Fragility of, 255.
Bromide of Potassium — Epilepsy, 269.
Bromide of Iodine, 458.
Brinsmade's Diaphoretic Powder, 458.
Bromide of Potassium, 483, 619, 683.
Bowels, 490.
Bell and Gaillard, 491.
Belladonna Poisoning, 531.
Bronchitis, Infantile, 597.
Burns, 745.
Bismuth in Dysentery, 599.
Beef Tea, Cream, 599.
Bright's Disease, Lime Water, 604.
Bodies, Preservation of, 605.
Biliary Calculi, 655.
Blood Stains, 744.

Cancer of Stomach, 11.
Chemistry Applied, 89.
Contagion of Phthisis, 98, 424.
Cystotomy, 137.
Carbolic Acid Solutions, 184.
Confectionery, Poisonous, 214.
Colloid of the Brain, 215.
Chorea, 223.
Curare, Physiol. and Therap. of, 252.
Cancer Curers, 296.
Carbolic Acid. 299, 425, 491, 600, 743.
Cinchona Barks, Chemistry of, 389.
Crime, Infection of, 393.
Caution to Prescribers. 396.
Chloroform, Death from, 421.
Calabar Bean, 427, 605.
Criminal Carelessness, 487.
Croton Oil, Poisoning by, 486.
Chinese Physicians, 542.
Carr, Prof. E. S., 549.
Constipation in Old Age, 552.
Clavicle, Fracture of, 556.
Chloral, 588, 618.
Cystitis, Chronic, 601.
Calabar Bean in Epilepsy, 601.
Catarrh, Chronic, 603.
Cholesterine, 608.
Colotomy, Edwards, 614.
Chancres, 678, 680.
Catarrh, Common, 678.
Croup, 679.

Dyes, Poisonous, 224.
Diphtheria, Sol. of Memb., 254.
Decapitated Subjects, 397, 429, 461.
Delirium Tremens, 743.
Dispensing Medicines, 458.
Diphtheria, Treat., 556, 678.
Diarrhœa, Infantile, 597, 680.
Dental Splint, 666.
Drowning, (Howard,) 681.
Digitalis, External Use, 684.
Dental Hæmorrhage, 708.

Electricity in Hysteria, 48.
Elimination of Saliva, etc., 92.
Earle, Dr., Case of, 181.
Epilepsy Cured by Pregnancy, 207.
Electricity, Medical, 239, 284.
Earth Closets, 514.
Epilepsy, Brown Sequard, 602.
Ecraseur Amputation, 631.
Embolism of Heart, etc., 742.

Faunius, Prolapsus, 256.

Fees, Med. Coll., 447, 452.

Gelseminum, 16.
Graduates, Pr. M. C., 1868–9, 95.
Grafts, Animal, 123.
Gonorrhœa, 223.
Gums, Diseased, 598.
Glandular Dis., Iod. of Am., 601.
Goitre, 602, 603.

Heat, Animal, 217.
Haven, E. O., D.D., 453.
Hydrophobia, 506.
Hospital Reports, 508, 565, 637.
Hæmorrhage, Pathogenesis, 533.
Hernia, Morphia Subcutan., 552.
Hip-Joint, Resection, 588.
Headache, 597.
Hæmorrhoids, 600.
Hæmoptysis, 681.
Hospital for Insane, State, 736.

Iron, Solution Nitro Sulph., 41.
India-Rubber Sponge, 189.
Insane, Ears of, 216.
Iron, Syrup. Iod., 222.
Impostor, 364.
Intemperance, Parrish, 638.
Impetigo, 680.

Kidneys, Tubercular, 208.

Legislation, Medical, 60, 156, 210.
Laryngoscopy, New Method, 123.
Locations, 455.
Library Am. Med. Assoc., 549.
Lead Poisoning, 553.
Lactic Acid in Croup, 748.

Monstrosity, 15, 255, 256, 704.
Mental Influence in Disease, 66.
Motor Nerves, Periph. Term., 97.
Malformation, Eye and Ear, 163.
Milk, 748.
Murder, A New Defence, 178.
Malaria in Italy, 213.
Malarial Hæmaturia, 740.
Medicated Vapors, 554.
Metrorrhagia, 554, 587, 589.
Materia Med., Indian, 733.

Nerves, Regeneration of, 281.
News Items, 455.
Names Again, 489.
Nerve Cells and Axis Cylinder, 493.
Nerves, Cutaneous Terminations, 562.
Neuralgia Plaster, 601.
Navy, 666.
Nipples, Sore, 679.

Oxygenation (Super) of Blood, 116, 139, 380, 441.
Opium, Therap. Use of Fumes, 129.
Obituary Notices, 212, 363, 428, 492, 550.
Opium and Pigeons, 222.
Organic Life, Dynamics of, 232.
Opium Culture, 298.
Opium, Poisoned by, 720.
Ovariotomy, 482, 697.
Ozone, 598, 670.
Orchitis, 606.
Oysters, 608.
Ozæna, 678.
Obstetrical Case, Brooks, 717.

Prunus Virginiana, 50.
Placenta Præmatura, 84.
Placenta Prævia, 717.
Puerperal Metro-Peritonitis, 110.
Parturition, Difficult and Tedious, 158
Partnership, Advertisements for, 179.
Paper Collars, 180.
Pathogenesis — Infusoria, 193.
Poisons — Mode of Action, 216.
Pepsine, 221.
Parasites, 222.
Pessaries, Medicated, 222.
Picrotoxin, 395.
Parasitic Diseases, 426.
Priapism, 459.
Pharmacopœia, Universal, 487.
Perspicuity, Professional. 513, 592.
Pancreas, Iutim. Struct., 532.
Pacinian Corpuscles, 557.
Pamphlets Acknowledged, 584.
Publisher, Change of, 590.
Photographs, Med., 591.
Publishers' Notice, 594.
Parker, Willard, Prescript., 606.
Pertussis, 679.
Prescriptions, Writing, 738.

Rush Med. College, 61, 64, 95, 445.
Reflex Actions and Electric Currents, 133.
Rheumatism, Acute, 278.
Reversio, 708.

Semen of Old Men, 94.
Sunstroke, 121, 602.
Shoulder Joint, Amputation, 134.
Snake Bites, 183.

Sea Sickness, 185.
Spain, Med. Education in, 213.
Styptic Paper, 223.
Strychia Antidote, 224.
Sterility, Microscope in, 411.
Spine, Dislocation of, 446.
Sponge Tents, 553.
Solvents for Croupous Membrane, 555.
Stomatitis, Gargle, 599.
Styptic Collodion, 605.
Sulpho Phenic Acid, 606.
Starchy Food, Infants, 608.
Sleeplessness, Infantile, 619.
Syphilis and Vaccination, 632.
Somnambulism, 650.
Specialties, 670.
Schools, Inj. Infl. on Health, 671.
Sycosis, 678.
Scabies, 680.
Schœppe, Dr., Case of, 745.

Tobacco Denicotinized, 87.
Tubercle, Pathogenesis, 98.
Throat Diseases, Practical Point, 181.
Tænia from Raw Meat, 214.
Tænia Solium, 721.
Tendo Achillis, 224.
Tetanus Traumatic, 483.
Tic Doloureux, 525.
Trichinitis, 743.
Trephining Skull, 586, 598.
Turpentine, Med. Ap., 619.
Tumor, Fib. Cell., 707.
Toothache, 678.
Trismus Nascentium, 679.
Therapeutic, 683.
Transfusion of Blood, 705.

Uterus, Displacements of, 20.
Uterus, Inversion of, 668.
Urethra, Rupture and Operation, 137.
Uterine Hæmorrhage, Iodine Injection, 482.
Uterine Polypus, 530.

Vaccination, 298, 540, 589, 620, 632.
Vomiting of Pregnancy, 553.
Vaginitis from Lead Poisoning, 555.
Variola, Prevent. Pitting, 589.
Varicose Ulcers, 681.
Vaccinal Syphilis, 696.

Wombless Women, 186.
Waukesha Springs, 203.
Western News Company, 297.
Wound, Lacerated and Contused, 375.
Watermelon in Dysentery, 590.
Water, 599.
Warts, 600.
Wounds, Antiseptic Treat., 610.
Womb Diseases, Norcom, 712.
Women, 737.

MEDICAL SOCIETIES:
Carroll Co., 31, 176, 449.
Wisconsin State, 55.
Chicago, 126, 359, 406, 448, 506, 535.
Alumni, Rush, 152.
Am. Med. Association, 154, 173, 333, 365.
Illinois State, 176, 347.
Microscopic, 885.
Medical Journalists, 387.
Brainard (Indiana), 396.
North Iowa, 408.
Minnesota State, 450.
Fillmore Co., Minn., 451.
Am. Pharmaceutical Assoc., 595.

CORRESPONDENCE:
G. Newkirk, M.D., 24.
A. W. Bosworth, Paris, 102, 701.
E. S Carr, California, 105.
Baldwin & Nott, 257.
E. R Hutchins, Philadelphia, 373, 410, 704.

BOOK NOTICES:
De la Cantharide, etc., 25.
Surgeon Gen. Report, 1868, 28.
Physiology and Hygiene, Dalton, 28.
Hand-book of Vaccination, 29.
The Opium Habit, 29.
Lectures on Fever, Hudson, 29.
Prin. and Prac. Med., Paine, 63.
Obstetrics, Longshore, 63.
Dis. of Urinary Organs, Thompson, 63.
Chronic Bronchitis, Greenhow, 63.
Laryngoscope, Mackenzie by Cohen, 64.
Wythe's Dose and Symptom Book, 64.
Cleveland's Lexicon, 64.
Ophthalmic Surgery, Phillipps, 128.
Organic Life, McElroy, 157.
Uterine Therapeutics, Tilt, 158.

Archives Ophthalmology and Otology, 158.
Pathological Phenom. Generalized, 191.
Identity of White Corpuscles of the Blood with Salivary Pus and Mucous C., 191.
A History of the Medical Department of the University of Pennsylvania, 191.
Pennsylvania Hospital Reports—Vol. 2, 192.
A Treatise on Diseases of the Ear, etc., by Anton Von Trolsch, M.D., 192.
A Treatise on the Diseases of Infancy and Childhood, Smith, 192.
Syphilis and Local Contagious Disorders, Hill, 192.
The part taken by Nature and Time in the Cure of Diseases, etc., 192.
Damon on the Skin, 300.
Dis. of Women, Thomas, 300.
Catalogue Pathol. Museum Pennsylvania Hospital, 300.
Club-Foot, Lewis A. Sayre, M.D. 460.
Diseases of the Eye, Wells, 516.
Chemistry, Fownes by Bridges, 517.
Digestion, Pavy.
Lachrymal Affections, Arlt, 518.
Important Med. Works (Hurd & Houghton), 547.
Dis. and Surg. of the Mouth, Jaws and Associated Parts, Garretson, 578.
Chemistry, Odling, 579.
Electricity, Meyer by Hammond, 579.
Practical Medicine, Niemeyer, 579.
Hip-Joint, Bigelow, 581.
Surgery, Erichsen, 582.
Metrop. Board of Health, N. Y., 583.
Guide-Book, Florida, etc., 583.
Circular No. 2, Surg. Gen. U. S. A., 583.
Sleep and its Derangements, Hammond, 657.
Ophthalmic Surgery, Laurence, 663.
Urinary Fistulæ, Thompson, 663.
Surgery of the Cervix, Emmet, 664.
Intra-Ocular Tumors, 723.
Dis. and Inj. of Eye, Lawson, 724.
Riley's Materia Medica, 724.
Hoppe, Percussion, etc,, 725.
Physicians Problems, 725.
Man in Genesis, etc., 731.
Transactions Am. Med. Association, 734.
Elmer's Hand-Book, 1870, 735.

**RENEW YOUR SUBSCRIPTIONS.**

☞ ALL SUBSCRIPTIONS expire with the present Number.
☞ Send $3 00 PROMPTLY for the new year, commencing with the next number.

### INDEX.

☞ N. B. By an unaccountable mistake, the numbers for April and May were paged alike. For convenience of reference, the duplicate paging for May in the Index will have affixed the italic *a*.

Abscess in Long Bones ........ 18
Aneurism, Femoral, Operation ........ 36
Alcohol, Absinthe, etc. *Trans* ........ 38
Acid, Phosph. Dilut ........ 57
Aiken, S. C., Climate, etc ........ 59
Albumen, Andral ........ 115
Alumni Meeting R. M. C ........ 123
Ancient San. Regulations ........ 126
Address, Alumni, Ames ........ 145
Aortic Aneurism ........ 155
Alcoholized Sponge ........ 187
Alcohol, Test for Water in ........ 190
Anus, Abnormal Situation ........ 208
Aneurism, Popliteal, Spontaneous ........ 218
American Med. Association ........ 256, 367
Aphthae, Formula for Lotion ........ 251
Aneurism of Orbit ........ 199*a*
Akephaloid Monster ........ 205*a*
Autoplasty ........ 219
American Builder ........ 231*a*
Arts, The, Chicago ........ 232*a*
Albany Med. College ........ 236*a*, 685
Ancient Norse Surgery ........ 351
Alphos Circinatus ........ 401
Aphasia and Thrombosis ........ 408, 467
Atropia *vs* Morphia ........ 471
American System, The ........ 503, 625
Apostolic Succession ........ 560
Antiseptic Fluids ........ 552, 565
Abscess, Peri Uterine ........ 573
A Noble Benefice ........ 624
Antiseptic, Local and General ........ 626
Antidote, Snake Poison ........ 632
Asthma, New Remedy ........ 637
Alimentation in Health and Disease ........ 669
Arrowhead in Lung ........ 676
Artificial Anus, Gunshot Wound ........ 676
Acid Mixture ........ 698
Arsenic Hypodermically ........ 701
Anatomical Wills ........ 701
Artificial Limbs for Soldiers ........ 704

Burns, Glycerine and Carbol. Acid ........ 34
Bright's Disease, Pamphlet Notice ........ 50
Bath, Turkish ........ 82, 201
Blood in Disease, Andral ........ 112
Bile Injected into Veins ........ 219
Butterfield, Wm., M.D., Note ........ 243*a*
Belladonna Poisoning ........ 330
Biliary Calculi ........ 331
Blood, Red Corpuscle ........ 350
Burns, New Remedies ........ 439
Bladder, Tumor Removed ........ 454
Bladder, Horse Shoe Pessary in ........ 509
Bone, Abscess, Tibia ........ 594
Brain, Abscess, Trepanning ........ 705
Ball, A., Com ........ 713
Bone, Non-Union, Gross ........ 726
Beale and His Doctrines ........ 732
Bioplasm, Beale ........ 736

Caution ........ 64
Culbertson's Reply ........ 134, 210
Coroner's Inquests ........ 175
Chloral Hydrate. 183, 254, 464, 524, 572, 629, 646
Cataract, Operation ........ 222
Cactus Opuntia ........ 250
Chloroform Elixir ........ 250
Cerebro Spinal Meningitis ........ 202*a*, 610
Catalepsy Case ........ 208*a*
College Courant ........ 231*a*
Croup—Lactic Acid ........ 253*a*
Colloid, Broad Ligament ........ 321
Conjunctivitis Neonatorum ........ 394
Catarrh, Nasal ........ 416
Chloral and Chloroform ........ 440
Cancrum Oris ........ 441
Cell Theories ........ 449
Chorea, Hydrate Chloral ........ 464
"Country Members" ........ 509
Correspondents, To ........ 561, 563, 621
Cell Theories, Huxley and Virchow ........ 577
Clinical Cases, Surgery ........ 594, 653
Circumcision ........ 623
Chlorodyne ........ 626
Citric Acid—After Pains ........ 627
Cinchona Cultivation, India ........ 638
Carbolic Acid Mixture ........ 698
Camphor and Chloroform Mixture ........ 698
Cell and Functions, Beale ........ 734
Clinic, Surgical ........ 743
Cancer, Application ........ 766
Chinese Medication ........ 767

Ductus Com. Chol., Absence ........ 110
Diplomas for Sale ........ 123, 248
Diagnosis, Unique ........ 126
Dissection Wounds ........ 189
Dentition, Brom. Potassium ........ 190
Diphtheria, Epilepsy ........ 211
Diabetes, Milk Diet ........ 252
Dislocation Humerus ........ 204*a*
Dyspepsia, Acid, Infants ........ 243*a*
Diet in Surgical Cases ........ 254*a*
Dysentery, Acute ........ 405
Dysentery, Scorbutic ........ 599
Deaf Mutes Taught to Speak ........ 607
Diphtheria, Formula ........ 626
Diagnoses, Unhappy ........ 689
Diabetes, Acute ........ 763

Enuresis, Nocturnal ........ 52
Eye Shade, Improved ........ 178
Earth Closets ........ 178
Examination, Poetical Response ........ 190
Electricity, Parturition ........ 218

Epilepsy Case .......................... 222
Ergotine, Hypodermic .................. 25c
Epistolary, "Viator" ............ 207*a*, 611
Electric Currents, Ner. System, *Trans.* 211*a*, 331, 419
Electric Currents, Paris Letter ....... 220*a*
European Mail ......................... 232*a*
Emphysema from Parturition ........ 240*a*
Experts, Medical ................... 437, 510
Eye, Statistical Report, Holmes .... . 521
Explosive Pills ........................ 566
Erysipelas, Local Application.. ...... 627
Epileptiform Seizures ................ 697
Ectropion Vesicæ, Maury ............. 727
Expectoration Fœtid .... ............. 764
Erigeron Tinct. in Alv. Hæm.......... 768

Fibrin, etc., in Disease, Andral......... 112
Francis, Dr., Portrait.................... 126
Fashion, Perils .... ....... ........ 188
Fever, Relapsing .... ................. 223
Fistula, Ancient Treatment............. 255
Fistula, Chisholm ... ................. 255
Flies, Malignant Pustule .............. 221*a*
Fees, Life Insurance Examination..... 437
Fissura Ani ... ....................... 567
Febrifuge Mixture...................... 627
Fistula in Ano (Edwards) ............. 636
Food in Nervous Diseases......... .. 662
Fire Proof Buildings .................. 686
Febrifuge Formulæ................. 695, 697
Freedom of Opinion..................... 762

Gross, Prof., Portrait...... ........... 63
Graduates Rush Medical College (1870) 121
Gastric Juice—Cancer .................. 249
Gout, Old Remedy ...... ............. 237*a*
Graduates 1870............ ............ . 352
Galvanized Iron Pipe .................. 634
Gangræna Genitalia in Infancy ........ 641
Gestation Protracted .................. 701

Hospitals, A word about .... 61, 248, 239*a*
Hermaphrodism, *Trans*........ ....... 156
Hiccough, Musk in... ........ ...... 205
Howard University ................. ... 251
Humerus, Dislocation ................. 204*a*
Harper's Magazine..................... 231*a*
Hip Joint, Subcutan., Operation..... 256*a*
Hair Restorer, Death from ............ 472
Helonias Dioica........................ 509
Heart, Fatty Degeneration ............. 624
Hermaphrodidsm ....................... 682
Hospital Cook County, clinical cases .. 690
Hyd. Chloral--Tetanus............ .... 709
Hip Joint, Resection................... 743
Humerus, Old Disloc., Reduction...... 744
Homœopathic Advertisement.......... 762
Hair Dye.............................. 768

Ileum, Strangulation .................. 32
Imaginary Cases ...................... 35
Iliac, External Ligation....... ........ 36
Ingratitude ....... .......... ... .... 63
Influenza, Treatment .................. 253
Infantile Mortality, Paris ............. 221*a*
Iodoform Suppositors ..... ........... 241*a*
Infants, Acid Dyspepsia............... 243*a*
Iodine in Intermittent.................. 256
Iodoform ........................ 566, 635
Indian Medicine ...................... 687
Ice in Aneurism ............ .......... 700
Inebriate Asylum, New York ......... 703
Innervation, Irregular ................ 741
Incremation in War ................... 768

Joints, Inc. and Punct. Wounds ...... 196*a*

Kerosene ....... ....................... 187
Kidney, Extirpation .......... 189, 252, 255

Life, Physical Basis ... 6, 134, 210, 215, 388 713, 716
Lupus Galvano Caustic ................ 253
Lactic Acid—Croup .... ............... 253*a*
Longevity—Oil ........................ 256
Little Corporal ....... ................. 502
Leucorrhœa, Brom. Pot ............ .. 661
Larynx, Removal entire ................ 702

Musk in Hiccough...................... 205
Monster, Akephaloid ................. 205*a*
Medical Society, Illinois State.... 242*a*, 356
Medical Society, Brainard .. ........ 243*a*
Make Haste Slowly.... ... ............ 352
Medical Teachers, Convention of.. 353, 507
Medical Society, Indiana.......... 367, 677
Magnetic Springs .... .......... 438, 622
Medical Education........ ............ 503
Muriatic Acid in Fever .. ....... .. 507
Medical Society, North East Indiana .. 510
Melancholy, Erotique.................. 559
Modern Miracle ....................... 561
McFarland, Dr. A...................... 562
Mineral Acids—Tuberculosis.......... 564
Marriage of Literary Men.... ........ 564
Mercurial Extinction . ................ 565
Medical Society, Esculapian........ .... 573
Menstruation, Non-Ovarian............ 583
Mistura Cretæ......................... 625
Mineral Spring, Portage, Mich ........ 656
Medical Society, Nebraska . ......... 675
Medical Society, Pennsylvania......... 680
Medical Society, New Jersey........... 682
Malarial Poisoning .................... 698
Microscope, High Powers ............. 733

Nymphomania . ....................... 176
News Items... 176, 249, 237*a*, 507, 564, 621
Nerves, Spinal, Br. Sequard ........... 253
Nervous System, Elect. Cur., *Trans*.. 211*a*
Nephrotomy ........................ 255*a*
Nævus, Treatment..................... 565
Necrosis, Wax Plug...... ... ..... .. 595
Nervous Disease, Food in............. 662
News Items, etc .... .................. 763
Nitro-Glycerin Power ................. 764

Obituary—J. R. Hamill, M.D.. ........ 64
Obituary—John Murphy, M.D........ 241*a*
Obituary—J. S. Hildreth, M.D.......... 512
Obituary—James Copland, M.D., etc .. 629
Obituary—Charles H. Ray, M.D ...... 702
Orbit, Aneurism of.................... 199*a*
Ontology in Medicine.... ............. 235*a*
Orchitis ....... ..................... 241*a*
Ossification, Extensive ................ 442
Owen, J. E., Com .. .................. 725
Œsophagus, Strict., Gastrotomy....... 728
Ozone ................................ 764

Placenta Prævia..... ............ ... 29
Poisoning—Sulph. Copper.......... .. 30
Puerperal Convulsions ......... .. 85, 684
Pharmacist ........................... 128
Paraplegia, Br. Sequard, Pavy ...... . 186
Paste, Recipe.. ...... ................ 187
Popliteal Aneurism, Spontaneous...... 218
Pneumonia, Tartar Emetic .. . .... 221
Puerperal Fever in Cook Co. Hospital 193*a*
Phosphate of Lime, Sulphited ....... 255*a*
Phenic Acid in Variola ................ 343

Polypus, Uterine, large ................ 385
Peritonitis, Traumatic ............. ... 397
Pertussis, Management ................ 441
Pleurisy, Purulent ...... .............. 481
Pharmaceutical Association, American 511
Positivism, Superfine .................. 567
Poisoning—Large Draughts Water ... 639
Palato-Pharyngeal Tumor ......... 653, 761
Poisoning Case, Atlee ....... ....... 696
Phosph. Lime—Profuse Perspiration.. 701
Publishers' Notice .... ... ........... 760
Pregnancy, Intra and Extra ............ 765
Polyscope ............................ 765

Rush Medical College ............ 63, 121
Responsibility, Editorial ... ....... .... 63
Retroversio Uteri, Jones ............... 193
Relapsing Fever ....................... 223
"Railway Spine" ... .... ........... 240*a*
Rabies .. ............................ 439
Rip Van Winkle, *Poem* .......... . 443
Rotary Movements ............ .. 486, 525
Ruppaner—Sayre ........... ......... 628
Rhinoplasty ...................... ... 725

Strangulation—Ileum ................ 30
Syphilis, Cases, Norcom ............... 92
Sterility, Microscopic Observations ... 101
Scientific American ................ ... 127
Shoulder Joint, Amputation, etc., Hess 207
Skull, Fracture, "Viator" ........... 207*a*
Spotted Fever in Virginia in 1820 ..... 237*a*
Simpson, Sir J. Y., Death .............. 350
Scarlatina, Heroic Treatment ... ...... 388
Sulphites in Phleg. Ang . ............. 539
Sunstroke ........................... 551
Speculum, Nott's ...................... 568
Syphilization ....................... 569
Stomach Pump ............... 571, 572, 624
Spine, Fracture ........... .. ... .... 600
Scabies, Bals. Peru ........ ....... 623
Strychnia in Cholera .................. 623
Snake Poison—Antidote. .. .......... 632
Scalping Case ......................... 675
Scarlatina ......................... 683, 694
Stricture, Urethral, Gross .............. 727
Spinal Affect., Suspension ........... .. 745
Squibb's Tinct. Op. Co.... . ........ 765
Small-pox in Paris ..................... 767

Scarlatina, Temperature ............... 767
Small-pox in Russia ..................... 768

Taenia Solium ...................... .... 27
Trichina Spiralis, History, etc . ..... 65
Temperature in Typhoid ....... ... 104
Temperature in Disease, Andral ..... . 112
Trichiniasis, Reed . ............ ......... 130
Transfusion ............ .......... .. 178
Tents, Alcoholized Sponge ............ 187
Tartar Emetic, Pneumonia ............ 221
Tubular Pregnancy ..................... 328
Tardieu, Prof ........ ............... 349
Thrombosis and Aphasia .. ... . 408, 467
Tympanites, Intest. Punct.. .......... 439
Thermometer in Diagnosis ........... . 460
Typho-Enteric Mixture .......... .... 627
Traumatic Tetanus ..... .............. 709
Tobacco—Drugging .......... ........ 764

Uterus, Natural Supports ....... ... . 1
Urine, Incontinence . ............. . 52
Urea in Febrile Disease, Andral ....... 116
Ubique Gentium? ..................... 233*a*
Uterine Polypus Extirpated ........... 385
Underbidding ........................ 505
Uterine Fibroid, Pariet. Remov ....... 513
Uterus, Malignant Disease ......... .. 553
Ureth. Stricture, Forcible Dilatation ... 597
Urinals, Public ........................ 766

Venereal Excess .................. .... 23
Variola and Vaccination, Paris ......... 341
Verat. Virid., Heroic Use ............ . 391
Vagina, Double ........................ 485
Veratrum in Opium Poisoning ........ 566
Vertebra, Dorsal Disloc., Partial Recov. 575
Vomiting, Obstinate .................. 627
Veratrum Viride, Therap ........... .... 716
Vaccination, Italian Army .............. 766

Womb, Plato ......................... 118
Western Monthly ................ 128, 689
Wheat Phosphates, Therap... ....... 251*a*
Woman's College ..................... 622
Who? .... .......................... 690
Women at Clinics .. ................... 731

Zircon Light ............ ............ 249

### BOOK NOTICES.

Diseases of Children, Vogel ........ ... 47
Wasting Disease of Child, E. Smith ... 48
Hypodermic Medication .............. 49
Obstetric Aphorisms, Swaym. ........ 119
Manual Clin. Med., etc., Tanner ...... 120
Clinical Medicine, Bennett ......... .... 172
Bellevue and Ch. Hospital Report, 1870. 172
Personal Beauty .......... ........... 173
Physiology, Flint ....... ............... 240
Tyson's Cell Doctrines ................ 241
Naphey's Modern Therapeutics ....... 242
Hall's Health by Good Living ......... 242
Diseases of Children, Meigs & Pepper 223*a*
Diseases of Eye, Williams ... ........ 224*a*
Chem. Examination Urine, Flint, Jr.. 224*a*
Disease of the Heart, Flint .... ...... 225*a*
Hand Book of Surgery, Packard ...... 225*a*
Preventive Obstacle, Bergeret ........ 226*a*
Indigestions, Chambers .............. 344
Membrana Tympani, Politzer ........... 345
Gentleman's Stable Guide ............... 346
Barnes' Obstetric Operations ...... 346, 428
Gray's Anatomy ................. 346, 435
Life of Galileo ........ ............. 346
Howe on Fractures .... ............. 347
U. S. San. Commission Memoirs.. 347, 436
Basham, Renal Disease ............ 347, 435
Fourth Annual Report of the Metropolitan Board of Health (N. Y.) ..... 495
Superstition and Force ..... .......... 496
Tanner's Practical Medicine ........... 549
Byford's Obstetrics .................. 550
Microscopic Manipulation, Suffolk .... 551
Medical Diagnosis, Da Costa .......... 618
Phy. Explo. Rectum, etc., Bodenhamer 619
American Dispensatory (Eclectic) ..... 616
Shaw's Case Book ..................... 688
Prescription Record ... .. ........... 690
Hand Book of Microscopy, Richardson 754
Path. and Treat. Venereal Dis., Bumstead ................ . ....... 755
Pract. Anatomy, Manual of Dissections, Heath .................. .......... 756
Physiology and Hygiene, Hutchison ... 757

### PAMPHLETS.

Bright's Disease, Lewis ................ 50
Nocturnal Enuresis, etc., Snelling..... 52
Acid. Phosph. Dil., Andrews .......... 57
Mortality in Armies, Newman......... 61
Braith. Retrospect, P. L. X............ 120
American Journal of Obstetrics ....... 120
Various Monographs........ 173, 174, 228*a*
Sedative action of Calomel, Lente..... 227*a*
Physical Basis of Life, Huxley. ....... 230*a*
Reduction of Dislocations, Green .... 347
Archives Ophthal. and Otol.,V. 1, No. 1. 348
Various ............................ 436, 559
Protoplasm, Stirling........... .. .... 498
The Bromides, McElroy. .............. 498
Treatment of Croup..... .............. 499
Third An. Rep. Brainard Free Dispen... 500
Woman's Medical College, N. Y...... 501
McFarland—Hammond .... ....... 501
N. Y. State Inebriate Asylum........... 501
Literarischer Monatsbericht........... 502
Examination of Urine, Legg........... 551
Sunstroke, Van DeWarker ............ 551
Benzole and Arsen., Clapham ....... 552
Malignant Disease of Uterus, Barker.. 553
Reform, Medicine and Morals, No. 3.. 558
Origin of Diabetes, Lusk .............. 675
Medical Times, New Journal ....... .. 686
Portraits Diseases of Skin, N. Syd. Soc. 757
Visiting List, Lindsay & Blakiston..... 758
Photographic Review.................. 759

### CORRESPONDENCE.

Foreign, Bosworth....... 111, 115, 219, 341

"Viator" ............................ 207*a*

